# Impacts of Graded Levels of Metabolizable Energy on Growth Performance and Carcass Characteristics of Slow-Growing Yellow-Feathered Male Chickens

**DOI:** 10.3390/ani9070461

**Published:** 2019-07-19

**Authors:** K. F. M. Abouelezz, Y. Wang, W. Wang, X. Lin, L. Li, Z. Gou, Q. Fan, S. Jiang

**Affiliations:** 1 Guangdong Key Laboratory of Animal Breeding and Nutrition/Guangdong Public Laboratory of Animal Breeding and Nutrition/The Key Laboratory of Animal Nutrition and Feed Science (South China) of Ministry of Agriculture/State Key Laboratory of Livestock and Poultry Breeding/Institute of Animal Science, Guangdong Academy of Agricultural Sciences, Guangzhou 510640, China; 2 Department of Poultry Production, Faculty of Agriculture, Assiut University, Assiut 71526, Egypt; 3 Academy of State Administration of Grain, Beijing 100037, China

**Keywords:** energy requirement, meat quality, growth performance, slow-growing broilers, nutrient deposition

## Abstract

**Simple Summary:**

Inadequate feed inhibits the potential performance of birds, and giving birds excess nutrients or levels higher than the requirement reduces production profits and may lead to negative effects on performance. Although recently there has been an expanding market worldwide for slower growing chickens to meet the consumer demand for a better tasting meat, little effort has gone into optimizing their dietary nutrient levels. Using fiv e different dietary energy levels, this study evaluated the optimal requirement of dietary energy for maximal growth rate, feed:gain ratio, meat quality indices, and blood metabolites of a Chinese yellow-feathered breed.

**Abstract:**

A dose-response study was conducted to investigate the metabolizable energy (ME) requirement for Lingnan chickens from 9 to 15 weeks of age. One thousand two hundred 8-week-old slow-growing yellow-feathered male chickens were allotted to five dietary ME levels (2805, 2897, 2997, 3095 and 3236 kcal/kg). The results revealed that the daily metabolizable energy intake increased (*p* < 0.01), whereas the feed intake and feed:gain ratio decreased linearly (*p* < 0.01) with the increment in dietary ME level. The final body weight and daily gain of the highest ME treatment tended (*p* > 0.05) to be greater than those obtained with the lower ME levels. The fat content in breast muscle showed a quadratic response (*p* < 0.05) to the increase in dietary energy level. The shear force values of breast muscle in the 2897, 3095 and 3236 kcal/kg treatments were lower (*p* < 0.05) than those of the 2997 kcal/kg treatment. In conclusion, among the tested ME levels, 3095 kcal/kg was adequate for feed intake, shear force, and plasma uric acid, and 3236 kcal/kg tended to increase the body weight, body gain, and feed conversion ratio of Lingnan males between 9 and 15 weeks of age; further studies are still required for testing higher levels.

## 1. Introduction

In poultry production enterprises, feed cost accounts for around 70% of the total costs involved in production. Among the different feed-stuffs used in formulating poultry diets, the source of dietary energy resources is a major cost; 70% of the total poultry diet content are energy sources. Optimizing the dietary energy level, therefore, is important for lowering the feed cost per unit of poultry products [1]. Increasing dietary energy level provides fundamental benefits in the feed conversion ratio (FCR) of broilers, mostly by decreasing feed consumption [2,3,4]. On the other hand, using excessive energy or a level higher than the requirement can increase the deposition of undesirable abdominal fat in broiler carcass, considered to be an economic loss as it is often counted as a waste product [2]. The dietary energy can be optimized for both growth performance and for enhanced meat quality. Dietary nutrient levels alter meat color, energy content, and histological makeup as well as the metabolic characteristics of broiler muscles [5,6,7].

The optimal dietary energy for broilers has been estimated in several previous studies [8,9], but existing data require verification for modern genotypes [10]. In contrast, little effort has gone into optimizing the dietary energy level for slow-growing meat-type chickens. Recently, there has been an expanding market worldwide for slower growing meat-type chickens, giving them a place in contemporary production. This is mainly to meet the consumer demand for better tasting meat and for fulfilling organic production conditions [11], as well as avoiding some problems with the fast-growing broilers, such as sensitivity to environmental conditions, leg problems, metabolic failure, ascites, sudden death, and an increased mortality rate occurring during the finishing phase [12,13,14]. This relatively new interest in slow-growing meat chicken breeds is increasing worldwide, though it is associated with higher costs of production [11].

China is the second-largest global producer of chicken meat, almost half of which is from Chinese yellow-feathered breeds [15]; Chinese annual production of such breeds exceeds four billion birds. The distinct flavor and favorable color of the meat are highly desired by local consumers in China and in neighboring countries [16]. There are three types of such chickens [17], broadly classified as fast- (marketable around 8–10 weeks, 1.47–2.30 kg weight), medium- (marketable 9–14 weeks, 1.00–2.27 kg weight), and slow-growing (marketable 12–25 weeks, 1.06–1.88 kg weight). The increasing commercial importance of these indigenous birds means that comprehensive work is needed to improve their feeding standards. As the dietary energy requirement for slow-growing yellow broilers has not been estimated or optimized, the present study has evaluated the effects of different dietary ME levels on growth performance, blood biochemical variables, carcass quality, body composition, rate of energy deposition, and fat content in breast and thigh muscles.

## 2. Materials and Methods

### 2.1. Chickens, Diets and Management

The experimental conditions were approved by the Animal Care and Use Committee of the Institute of Animal Science, Guangdong Academy of Agricultural Sciences, China, with the approval number GAASISA-2015-011. The yellow-feathered male chickens (Lingnan breed, a meat-type breed that originated in South China) were obtained from a commercial hatchery (Guangdong Wiz Agricultural Science and Technology Co., Guangzhou, China) and were raised from day 1 to 8 weeks of age on a common, typical diet, provided ad libitum. One thousand two hundred birds were weighed at 8 weeks of age and randomly allocated to 30 equally-sized (4.55 m^2^) floor pens of 40 birds, having a similar average body weight (BW) (771.25 ± 10.23 g). Five dietary treatments, each with six replicates, consisting of graded metabolizable energy (ME) levels (2900, 3000, 3100, 3200 and 3300 kcal ME/kg, calculated), were pelleted and provided ad libitum, as was water. These experimental diets (Table 1) were formulated to provide the nutrient requirements of Chinese yellow-feathered broilers [18], except for the ME level. The gross energy of the diets was analyzed according to the guidelines of Association of Official Analytical Chemists [19], and the ME was determined and calculated according to the methods and the equation of Jiang et al., [20], which showed 2805, 2897, 2997, 3095 and 3236 kcal/kg, respectively. The 2997 kcal/kg was considered to be the control dietary energy level diet according to the previously determined value [18]. The birds were raised under artificial lighting providing 18 h light:6 h dark. Relative humidity and average room temperature were approximately 70.0% and 18 °C throughout the 7-week experimental period (9–15 weeks of age).

### 2.2. Growth Variables

The amounts of provided and refused feed were measured weekly on a replicate basis to calculate the average daily feed intake (ADFI), including adjustments for any dead birds. Mortality of birds was recorded daily. The initial BW, final BW (FBW), average daily body weight gain (ADG), and feed:gain ratio (g/g) (FCR) were measured on a per replicate basis. Metabolic BW was calculated according to the following equation: [(Initial weight + final weight)/2] ^0.75^.

### 2.3. Sampling

At 15 weeks of age, after 12 h of feed-withdrawal, blood samples were collected in 5 mL heparinized tubes from the jugular vein of 12 birds per treatment (2/replicate) who had BW values within ± 10 g of the average; plasma was obtained by centrifugation at 1000*×* g for 15 min at 4 °C. The birds were slaughtered by approved methods for subsequent analyses. The right and left breast muscles were separately sampled, clear of observable connective tissues, and stored at −20 °C until analyses; the right breast muscle (*Pectoralis major* and *minor*) was sampled for meat quality determinations, and the left muscle was used in measuring the chemical composition.

### 2.4. Carcass Trait Determinations

Dressing percentage (bled and defeathered carcass weight (CW), including head and feet, expressed as a percentage of BW), semi-eviscerated (CW minus weights of trachea, crop, esophagus, intestine, pancreas, spleen, gallbladder, gonads, contents of the proventriculus, and gizzard lining, expressed as a percentage of BW), and eviscerated proportions (semi-eviscerated weight minus neck, head, liver, heart, gizzard, shank, abdominal fat, and proventriculus, expressed as a percentage of BW) were calculated. In addition, the relative weights of de-boned thigh muscle, breast muscle, and abdominal fat, expressed relatively to BW, were calculated following the methods of the Chinese National Poultry Breeding Committee [22]. The breast and thigh muscles were placed in polyethylene bags and stored at −22 °C until chemical analysis.

### 2.5. Meat Quality Determinations

Meat pH, color (a* redness, b* yellowness and L* lightness), and drip loss were measured following the methods of Jiang et al. [23]. Meat pH was measured in the major right *Pectoralis* using a portable pH meter (version HI8424; Beijing Hanna Instruments Sci. & Tech. Co., Ltd., Beijing, China). Three readings of breast meat color were scored with a Chroma Meter (CR-410; Minolta Co., Ltd., Suita, Osaka, Japan) at different, but consistent, locations on the medial side of each muscle then averaged. Meat color scores, using L* a* b* color scales, were measured; L* is lightness (0 = black to 100 = white), a* is green (a*) to red (+a*), and b* is blue (b*) to yellow (+b*). Drip loss was estimated following a method modified from Shang et al. [24]. Briefly, about 11 g (fresh weight) of regular-shaped muscle section (4 cm (length) × 2 cm (width) × 1.5 cm (thickness)) cut from the same location in the breast muscle was weighed and suspended on a steel wire hook, without any contact, in a plastic bag inflated with air and stored at 4 °C for 24 h. The muscle samples were re-weighed to evaluate the drip loss percentage, according to the following equation: [(initial weight − final weight)/initial weight] × 100%. Finally, the shear force of cooked breast muscles was measured according to the methods described by Jiang et al. [23], using an Instron Universal Mechanical Machine (Instron model 4411, Instron Corp, Canton, MA, USA).

### 2.6. Composition of Body, Breast and Thigh Muscles, and Deposition Rate of Energy and Protein

The frozen samples of left breast and thigh muscles were dissected into small pieces and finely homogenized in a blender at −10 °C. To measure the fat and protein content, deposition rate of energy and protein in the whole body, ten birds at the age of 8 weeks (at the beginning of this experiment) and two additional birds per replicate at the age of 15 weeks were selected and prepared according to the methods of Zhou et al. [25] and Xi et al. [26]. Contents of crude protein (CP), crude fat, and gross energy were analyzed according to the guidelines of AOAC [19]. The deposition rate of protein and energy was estimated following the methods of Xi et al. [26].

### 2.7. Blood Biochemical Variables

The plasma contents of uric acid (UA), triglycerides (TG), and cholesterol (CHOL) were measured colorimetrically using a spectrophotometer (Biomate 5, Thermo Electron Corporation, Rochester, NY, USA) and commercial kits (Nanjing Jiancheng Institute of Bioengineering, Nanjing, China).

### 2.8. Statistical Analysis

Each pen (replicate) served as the experimental unit. The effects of dietary ME levels were examined for each variable by ANOVA (JMP Ver. 8.0.2, 2009; SAS Institute Inc., Cary, NC, USA). Whenever significant effects of treatment were detected, Duncan’s multiple range tests were used to compare the means. Where appropriate, orthogonal polynomial contrasts were used to estimate the linear and quadratic effects of the increasing levels of ME, and a probability level of 0.05 was applied to test significance (SPSS software version 17.0.1., IBM, Armonk, NY, USA). Based on the key indices (ADFI, feed:gain ratio, daily ME intake, uric acid, fat content of breast muscle, and fat content of thigh muscle), quadratic regression equations were used to determine the optimal dietary ME requirement of Chinese yellow-feathered chickens [27]. Data are expressed as means for each diet.

## 3. Results

### 3.1. Growth Performance

Daily ME intake increased, but ADFI and FCR decreased as linear responses to the increment in dietary energy level. The FBW, ADG, metabolic BW, and mortality rate were not affected (*p* > 0.05) by the dietary ME level, but the 3236 kcal/kg diet tended to have greater FBW and ADG than those of the lower ME levels (Table 2).

### 3.2. Carcass Quality

The tested dietary ME levels did not exhibit any significant effect on the carcass quality traits in terms of dressing percentage, eviscerated and semi-eviscerated proportions, relative weights of breast muscle, thigh muscle, and abdominal fat (Table 3).

### 3.3. Composition of Body, Breast and Thigh Muscles

As shown in Table 4, the fat content in thigh muscle increased linearly (*p* < 0.05) with the increase in dietary energy level, whereas the fat content in breast muscle showed a quadratic response (*p* < 0.05), and the highest value was obtained with the level 2997 kcal/kg. The protein, fat and energy content in the whole body as well as the energy and protein deposition were not affected by the dietary ME level. According to the regression model, the highest fat contents (%) in the breast and thigh muscles were obtained with diets containing 3047 and 3135 kcal/kg (Table 5).

### 3.4. Breast Meat Quality

The results of breast meat quality as affected by the dietary ME level are shown in Table 6. The 2897, 3095 and 3236 kcal/kg diets resulted in lower shear force values (*p* < 0.05) than those of the control diet, and those of the 2805 kcal/kg diet had an intermediate value (*p* > 0.05). The pH value, drip loss percentage, and meat color grades L*, a* and b* did not differ (*p* > 0.05) among the tested diets.

### 3.5. Blood Biochemical Variables

The results shown in Table 7 indicated that plasma UA decreased linearly (*p* < 0.01) with the increase in dietary ME level. The CHOL and TG concentrations were not affected by the diets. The regression model indicated that the optimal plasma UA was obtained with a diet containing 3200 kcal/kg (Table 3).

## 4. Discussion

### 4.1. Growth Performance

The present study tested five dietary ME levels (kcal/kg), consisting of a control level (2997), two lower levels (2805 and 2897), and two higher levels (3095 and 3236), respectively. The increase in dietary energy level did not affect the FBW or ADG of the slow-growing male yellow-feathered chickens, but the highest ME treatment (3236 kcal/kg) tended to result in greater FBW and ADG than lower ME levels. The daily ME intake increased, whereas ADFI and FCR decreased as linear responses to the increment in dietary energy level. Birds typically eat to fulfil their energy requirement [11,28], which can explain the reduced ADFI for the highest two dietary energy levels. The improved FCR for the highest ME level is attributable to the reduced ADFI and the relatively increased ADG. Supporting results were reported by Infante-Rodríguez et al. [4], indicating that BW and ADG were not affected by the dietary energy; however, ADFI was reduced by a high caloric level, and FCR was improved with a moderate increase in dietary energy. The present results were consistent with the findings of Kim et al. [29], who observed a reduced ADFI with higher energy levels than the standard diet. Other studies differed [30], where final BW and FCR in broilers increased with higher energy levels (2994 to 3013 and 3081 to 3111 kcal/kg ME, starter and finisher phases). Contrary to the present results, Houshmand et al. [31] found that broilers fed low-energy diets were heavier than those fed a standard diet. The results of Ferreira et al. [3] showed that a dietary energy close to 3000 kcal/kg did not affect BW in broilers, but a lower caloric level reduced BW, and a higher caloric level reduced ADFI. These varied responses to dietary energy levels in previous studies result from using different genotypes at different ages. Kim et al. [29] reported different responses to energy level with different strains of broilers. The results obtained here with slow-growing Chinese yellow chickens favor the increase in energy level over the control (2997 kcal/kg) and lower levels; the highest calorie intake occurred with the most energy-dense diet. Touchburn et al. [32] similarly noted that caloric intake increased as dietary ME level increased.

### 4.2. Carcass Characteristics

For the slow-growing Chinese yellow chickens studied here, dietary ME level had no significant effect (*p* > 0.05) on the dressing percentage, eviscerated and semi-eviscerated proportions, relative weights of breast muscle, thigh muscle nor abdominal fat. Supporting results were reported by Infante-Rodríguez et al. [4], who tested dietary energy levels (2960 to 3160) close to those used here; there was no influence on carcass weight, breast, drumstick and thighs, wings and back fat weight or carcass yields. Rosa et al. [33] used diets with 2950, 3200 and 3400 kcal/kg ME, but observed no effect on breast weight, carcass yield or back fat, despite the increase in energy concentration depressing the yield of thigh and drumstick and increasing abdominal fat. A preliminary study of Waldroup et al. [34] indicated no effect of dietary caloric level on growth performance or abdominal fat, although a higher energy level increased dressing percentage in females, but not in males. The present results with male chickens are consistent with that of the latter study. Others [35,36], similarly, found no effect of dietary energy level on carcass yield and abdominal fat. In contrast, Zhao et al. [37] found that dressing percentage, breast and thigh muscles, and abdominal fat content were greater with dietary energy and lysine levels higher than those in their controls. Marcu et al. [38] reported an improved growth performance and carcass yield for the main cuts of broiler chickens fed diets with high energy and protein contents. The preponderant previous findings on the effect of dietary energy level in broilers were inconclusive, but the results of the latter two studies showed that increased dietary energy along with increased CP or amino acids may result in a higher meat yield.

### 4.3. Composition of Whole Body, Breast and Thigh Meat

In the present study, dietary energy level did not influence the protein, fat or energy content in the whole body, but the fat content in thigh muscle increased linearly with increased caloric level, whereas the fat content showed a quadratic response and the highest value was obtained with the 2997 kcal/kg diet. Other studies showed similar results, with dietary energy level having no effect on the chemical composition of broiler’s carcass muscles [39,40]. Ferreira et al. [3] indicated that using reduced dietary energy levels lowered the intramuscular fat in broilers. The present results are in partial agreement with those of Infante-Rodríguez et al. [4], who found that increased dietary energy had no effect on CP content in breast muscle, although the lower ME levels (2960 and 3040 kcal/kg) resulted in more lipids in breast meat than with higher caloric levels (3080 and 3160 kcal/kg). In another study, Marcu et al. [41] found that decreasing dietary ME level reduced CP and increased the lipid content of broiler breast and thigh muscles. The results here showed that the fat (Table 5) content in the whole body was not affected by the dietary energy. The latter results agree with our results, which suggest that increasing dietary energy content for broilers may not increase meat lipids in the thigh and pectoral muscles.

In commercial Ross 308 broilers, Rosa et al. [33] reported that increasing the dietary ME level reduced carcass CP and increased its lipid content. Marcu et al. [42] found that increasing dietary energy and protein levels increased breast weight and muscle mass, and reduced fat content, but reducing nutrient level decreased protein content and elevated fat content in pectoral muscle. The discrepant results could be attributable to using different strains in the previous studies. Díaz et al. [43] and Rosa et al. [33] reported different changes in meat quality and carcass composition among different genetic groups fed graded levels of dietary energy.

### 4.4. Breast Meat Quality

The color of raw broiler meat is highly affected by dietary nutrient factors [5]. Meat color is an important attribute for consumers; the greater a* score of meat indicates better meat quality and the lower L* and b* scores implies less pale meat. Boulianne and King [44] reported that pale fillets have higher L* and b* values, and a lower a* value than normal fillets. No available information could be found on possible effects of dietary energy level on breast meat color. The most important finding in the present study is related to the shear force measured on the breast muscle, which decreased in the 2897, 3095 and 3236 kcal/kg diets. Increased shear force is associated with increased connective tissue and decreased fat content in meat [45,46]. This implies a reduced content of connective tissue in the breast meat of birds fed the 2897, 3095 and 3236 kcal/kg diets. The measured fat contents for the 3095 and 3236 levels were consistent with this interpretation to some extent. The control level (2997) had relatively higher (*p* > 0.05) breast fat; however, it unexpectedly showed a high shear force value. The reason behind this increased shear force with this energy level is not clear, or it might imply a high content of connective tissue in this treatment. Low drip loss and shear force indicate higher meat quality. Higgins et al. [47] and Min and Ahn [48] reported that increased drip loss and decreased meat color a* score reflects lipid peroxidation, leading to loss of pigments and deterioration of meat quality. Drip loss, meat color, and meat pH were not affected here by the dietary caloric levels used.

### 4.5. Blood Biochemical Variables

The evaluation of blood biochemistry in poultry shows metabolic alterations due to a number of factors, such as the physiological status, feeding standards, weather change, genetic type, age, housing conditions, and exposure to diseases [49,50,51]. The modification of dietary nutrient concentration can initiate stresses that induce dramatic changes in blood biochemistry [51,52]. In the present study, the increase in dietary ME level led to a linear decrease in plasma UA concentrations, likely reflecting changes in protein catabolism in the body [52]. The plasma UA values obtained here were comparable to those of Wang et al. [53], with the same chicken breed. According to the regression model, the lowest plasma UA was obtained with the 3200 kcal ME/kg diet, suggesting therefore that this level was optimal or most adequate for the efficient use of protein. This confirms that high caloric levels (3236 kcal/kg) are more adequate than the lower levels, which led to higher plasma UA content. This is consistent with Rosebrough, McMurtry, & Vasilatos-Younken [54,55], who found that reducing dietary energy increased broiler’s plasma UA content, and partially agreed with Rezaei and Hajati [52], who found in broilers at 21 d of age that a 40% reduction in dietary nutrients increased the concentrations of plasma UA; in contrast, they also found reduced plasma concentrations of CHOL and TG.

## 5. Conclusions

The increase in dietary energy level showed some benefits, with lowered ADFI, FCR, plasma UA, and shear force; without any adverse effect on the other meat quality variables, i.e., meat yield, nutrient deposition, mortality rate, or abdominal fat content. Under the conditions of this study, the 3095 kcal/kg diet was adequate for the best feed intake, shear force, and plasma uric acid, and the 3236 kcal/kg diet tended to increase the body weight and daily gain and reduce the feed conversion ratio of Lingnan males between 9–15 weeks of age; further studies are still required for testing higher ME levels. The regression analyses revealed that the optimal dietary ME levels for plasma UA, fat content in breast muscle, and fat content in thigh muscle were 3200, 3047, and 3135 kcal/kg, respectively.

## Figures and Tables

**Table 1:** Composition and nutrient levels of the experimental diets (%, as fed basis).

Item	Metabolizable Energy Levels (Kcal/kg, Calculated ^2^)				
	2900	3000	3100	3200	3300
Ingredients					
Corn (yellow)	70.26	70.26	70.26	70.26	70.26
Soybean meal	16.23	16.23	16.23	16.23	16.23
Corn gluten meal	3.8	3.8	3.8	3.8	3.8
Soybean oil	1.05	2.25	3.44	4.64	5.83
Limestone	1.12	1.12	1.12	1.12	1.12
Di-calcium phosphate	1.10	1.10	1.10	1.10	1.10
Salt	0.30	0.30	0.30	0.30	0.30
Vitamin-mineral premix ^1^	1.00	1.00	1.00	1.00	1.00
L-Lysine HCL (78%)	0.27	0.27	0.27	0.27	0.27
DL-Methionine (99%)	0.09	0.09	0.09	0.09	0.09
Corn cob meal	4.78	3.58	2.39	1.19	0.00
Total	100.00	100.00	100.00	100.00	100.00
Calculated composition ^2^					
Metabolizable energy (Kcal/kg) ^3^	2805	2897	2997	3095	3236
Crude protein (%)	16.00	16.00	16.00	16.00	16.00
Calcium (%)	0.80	0.80	0.80	0.80	0.80
Crude fiber	3.07	2.59	2.12	1.64	1.16
Total phosphorus (%)	0.56	0.56	0.56	0.56	0.56
Non-phytate phosphorus (%)	0.37	0.37	0.37	0.37	0.37
Lys (%)	0.85	0.85	0.85	0.85	0.85
Met (%)	0.37	0.37	0.37	0.37	0.37
Met+Cys (%)	0.65	0.65	0.65	0.65	0.65

^1^ Supplied per kilogram of diet: VA 5000 IU, VD3 500 IU, VE 20 IU, VK 0.5 mg, VB1 2.4 mg, VB2 4.0 mg, VB6 3.5 mg, VB12 0.01mg, niacin 30 mg, D-calcium pantothenate 10 mg, folic acid 0.55 mg, biotin 0.15 mg, choline chloride 1200 mg, Fe 80 mg, Zn 65 mg, Cu 7 mg, Mn 60 mg, I 0.35 mg, Se 0.3 mg. The vitamins and minerals in the diet were supplied exactly as stated by the Ministry of Agriculture of the People’s Republic of China [18]. ^2^ Values were calculated from data provided by the Feed Database in China [21]. ^3^ Analyzed values.

**Table 2:** Effect of dietary metabolizable energy level on average daily metabolizable energy intake and the performance of Chinese yellow-feathered chickens from 9–15 weeks of age.

Variables	Dietary Metabolizable Energy Levels (Kcal/kg), Analyzed Content					SEM	*p*	Linear	Quadratic
	2805	2897	2997	3095	3236				
Final body weight (g)	1386.6	1375.09	1408.08	1385.39	1430.12	16.85	0.0629		
Average daily gain (g)	12.82	12.58	13.27	12.79	13.73	0.35	0.0629		
Daily feed intake (g)	78.15 ^a^	78.32 ^a^	77.59 ^a^	75.28 ^b^	76.06 ^b^	0.59	0.0005	0.000	0.002
Feed: Gain ratio (g/g)	6.10 ^ab^	6.24 ^a^	5.86 ^bc^	5.90 ^b^	5.56 ^c^	0.13	0.0025	0.001	0.002
Daily metabolizable energy Metabolizable energy intake (kJ/d)	219.21 ^d^	228.45 ^c^	232.53 ^bc^	233.52 ^b^	246.06 ^a^	5.69	<0.0001	0.000	0.000
Metabolic body weight (g)	188.25	189.04	189.79	188.60	190.71	1.98	0.5248		
Mortality (%)	3.33	4.16	5.12	6.67	4.17	2.06	0.6668		

Means within a row with different superscripts differ significantly (*p* < 0.05). SEM = pooled standard error mean. Metabolic body weight = [(Initial weight + final weight)/2] ^0.75^.

**Table 3:** Effects of dietary metabolizable energy level on the carcass quality of Chinese yellow-feathered chickens at 15 weeks of age.

Variables	Dietary Metabolizable Energy Levels (Kcal/kg), Analyzed Content					SEM	*p*
	2805	2897	2997	3095	3236		
Dressing percentage (%)	89.68	88.93	88.98	87.89	88.84	1.01	0.1116
Semi-eviscerated proportion (%)	83.10	82.80	82.31	82.01	82.16	1.27	0.5329
Eviscerated proportion (%)	68.99	68.51	68.74	67.63	68.05	1.01	0.2321
Breast muscle (%)	15.60	14.62	14.95	15.19	14.23	0.63	0.0906
Thigh muscle (%)	18.87	19.05	18.42	19.12	18.89	1.71	0.9304
Abdominal fat (%)	1.10	0.87	1.29	1.71	1.63	0.29	0.0889

SEM = pooled standard error mean.

**Table 4:** Effect of dietary metabolizable energy levels on the compositions of body, breast and thigh muscles, and deposition rates of energy and protein in slow-growing Chinese yellow-feathered chickens at 15 weeks of age.

Variables	Dietary Metabolizable Energy Levels (kcal/kg), Analyzed Content					SEM	*p*	Linear	Quadratic
	2805	2897	2997	3095	3236				
Composition of body									
Crude protein (%)	65.31	62.56	62.43	60.58	60.77	8.90	0.4481		
Fat (%)	22.73	25.06	24.19	25.85	27.14	7.95	0.5656		
Energy (kJ/g)	23.20	24.18	24.02	24.05	24.05	0.44	0.4839		
Intramuscular fat content (%)									
In breast muscle	0.94 ^b^	1.12 ^ab^	1.64 ^a^	1.37 ^ab^	1.31 ^ab^	0.056	0.0441	0.386	0.016
In thigh muscle	5.12 ^b^	5.56 ^ab^	6.86 ^a^	6.18 ^ab^	6.64 ^a^	0.36	0.0288	0.025	0.033
Nutrient deposition rate (%)									
Energy	12.71	14.82	14.80	15.48	15.18	0.85	0.0755		
Protein	25.03	25.58	26.21	25.01	26.88	1.41	0.2732		

Means within a row with different superscripts differ significantly (*p* < 0.05). SEM = pooled standard error mean.

**Table 5:** Dose-response regressions for Chinese yellow-feathered chickens fed diets with different metabolizable energy levels from 9–15 weeks of age.

Variable	Model ^1^	Regression Equation ^2^	Response ^3^	*p*	R^2^
Uric acid (mmol/L)	QP ^1^	y = 21.494x^2^ − 575.47x + 3984	3200	0.012	0.144
Fat content of breast muscle (%)	QP ^1^	y = −0.572x^2^ + 14.587x − 91.435	3047	0.016	0.142
Fat content of thigh muscle (%)	QP ^1^	y = −0.715x^2^ + 18.765x − 116.62	3135	0.033	0.116

^1^ QP = quadratic polynomial; QP model = Y = α + β × X + γ × X^2^, where Y is the response variable, X is the dietary metabolizable energy (ME), α is the intercept; β and γ are the linear and quadratic coefficients, respectively. ^2^ Regression equations obtained using the analyzed metabolizable energy in the diets (2805, 2897, 2997, 3095 and 3236 Kcal/kg). ^3^ The response was obtained by −β/(2 × γ).

**Table 6:** Effects of dietary metabolizable energy levels on the breast meat quality of slow-growing Chinese yellow-feathered chickens at 15 weeks of age.

Variables	Dietary Metabolizable Energy Levels (Kcal/kg), Analyzed Content					SEM	*p*	Linear	Quadratic
	2805	2897	2997	3095	3236				
pH	6.11	6.00	6.08	6.06	6.02	0.009	0.736		
Drip loss (%)	2.06	2.22	2.19	2.06	2.08	0.076	0.948		
Shear force (kgf)	3.06 ^ab^	2.49 ^b^	3.52 ^a^	2.31 ^b^	2.62 ^b^	0.001	0.023	0.241	0.487
Meat color									
L* value	55.54	55.99	55.91	54.01	55.96	1.649	0.399		
a* value	15.02	15.24	16.46	15.76	14.77	0.572	0.136		
b* value	20.16	20.54	21.98	19.19	21.58	2.278	0.279		

Means within a row with different superscripts differ significantly (*p* < 0.05). SEM = pooled standard error mean.

**Table 7:** Effects of dietary metabolizable energy levels on plasma variables of slow-growing Chinese yellow-feathered chickens at 15 weeks of age.

Variables	Dietary Metabolizable Energy Levels (Kcal/kg), Analyzed Content					SEM	*p*	Linear	Quadratic
	2805	2897	2997	3095	3236				
Cholesterol (mmol/L)	3.12	3.19	3.22	3.09	3.13	0.07	0.9687		
Triglycerides (mmol/L)	0.32	0.33	0.377	0.37	0.33	0.002	0.3375		
Uric acid (mmol/L)	197.00 ^a^	159.83 ^ab^	156.13 ^ab^	117.21 ^b^	134.79 ^b^	3.81	0.0109	0.005	0.012

Means within a row with different superscripts differ significantly (*p* < 0.05). SEM = pooled standard error mean.
